# Treatment Results in Serpiginous Choroiditis and Multifocal Serpiginoid Choroiditis Associated with Latent Tuberculosis

**DOI:** 10.4274/tjo.37630

**Published:** 2017-04-01

**Authors:** Merih Oray, Zaur Zakiev, Tülin Çağatay, İlknur Tuğal-Tutkun

**Affiliations:** 1 İstanbul University İstanbul Faculty of Medicine, Department of Ophthalmology, İstanbul, Turkey; 2 İstanbul University İstanbul Faculty of Medicine, Department of Pulmonary Medicine, İstanbul, Turkey

**Keywords:** Serpiginous choroiditis, multifocal serpiginoid choroiditis, latent tuberculosis, anti tubercular therapy, immunomodulatory treatment

## Abstract

**Objectives:**

To compare the results of systemic antitubercular therapy (ATT) and immunomodulatory therapy (IMT) in patients with serpiginous choroiditis (SC) or multifocal serpiginoid choroiditis (MSC).

**Materials and Methods:**

The clinical records of 28 patients with SC and MSC were reviewed. Patients were divided into 2 groups according to the treatment applied. Group 1 included 12 patients with MSC and 5 with SC treated with ATT and corticosteroid (CS); group 2 included 9 patients with MSC and 2 with SC treated with conventional IMT, interferon alpha-2a, and/or CS monotherapy.

**Results:**

In group 1, clinical remission was achieved in 12/12 MSC and 3/5 SC (total 15/17) patients with administration of ATT for 1 year. Two patients (1 SC, 1 MSC) had reactivation 2 and 7 months after cessation of ATT. Two patients with recurrence after completion of ATT and 2 patients resistant to ATT received IMT ± CS therapy. In group 2, clinical remission was achieved in 7/9 MSC and 2/2 SC (total 9/11) patients after 1 year of treatment. Recurrent inflammation was observed in 2 MSC patients 2 and 112 months after initiation of therapy, but responded well to local/systemic CS or IMT modification, and clinical remission was achieved in 7.8±4.3 months. Cumulative dose of CS was higher in group 2 (p=0.057). Nine of 12 MSC patients treated with ATT and 4/9 MSC patients treated with IMT achieved remission (p=0.203). One of 5 SC patients treated with ATT and 2/2 SC patients treated with IMT achieved remission (p=0.142).

**Conclusion:**

Although a statistically significant result could not be achieved in this small case series, our results suggest that ATT may be an appropriate first choice in the treatment of MSC associated with latent tuberculosis, and may be administered in patients with SC who are unresponsive to IMT.

## INTRODUCTION

Serpiginous choroiditis (SC) and multifocal serpiginoid choroiditis (MSC) are uveitic entities on the same spectrum but with different clinical morphologic features. SC is a chronic, progressive, recurrent, usually bilateral intraocular inflammatory disease of undetermined etiology. It is characterized by geographic spread in the form of serpentine infiltrates typically beginning in the peripapillary region and spreading toward the periphery, involving the retinal pigment epithelium and outer retinal layers.^1,2^ Multifocal serpiginoid choroiditis, also referred to in the literature as serpiginous-like choroiditis, serpiginoid choroiditis, multifocal serpiginous choroiditis, and amphiginous choroiditis, may present as multifocal progressive or diffuse choroiditis.^3,4,5,6,7,8^ Similar to SC, the condition has a chronic, progressive, and recurrent course; however, in contrast to SC, it is characterized by multifocal, irregular geographic lesions in the fundus, midperiphery, and periphery in addition to the juxtapapillary area, and ocular involvement is frequently unilateral.^1^

Gupta et al.^3^ first described in 2003 that MSC is clinically distinct from classic SC and is associated with tuberculosis (TB). Recent studies have shown that aqueous and vitreous samples obtained from MSC patients are positive for *Mycobacterium tuberculosis* DNA.^4,9^ While SC is recognized as an immune-mediated inflammatory disease, the *M. tuberculosis* bacillus has been shown to be the triggering factor in MSC.^10,11,12,13^ The etiopathogenesis and treatment of both diseases is still debated.

The aim of this study was to compare the results of systemic antitubercular therapy (ATT) and immunomodulatory therapy (IMT) in patients with SC or MSC associated with latent TB.

## MATERIALS AND METHODS

The medical records of patients diagnosed with SC or MSC at İstanbul University İstanbul Faculty of Medicine, Department of Ophthalmology between January 1995 and December 2013 were analyzed retrospectively. Demographic data such as age and sex, symptoms at presentation, duration of symptoms, systemic and ocular histories, and diagnosis and treatment received at other medical centers were analyzed.

Patients with clinical findings consistent with SC or MSC, purified protein derivative (PPD) test results over 15 mm and/or positive interferon-gamma release assay test (IGRA; Quantiferon-TB Gold, ELISPOT), no manifest signs of extraocular TB, and who regularly attended follow-up for 12 months and regularly took their medications were included in the study.

Thirty-four patients who were followed for less than 12 months, who did not attend follow-up or take their medication regularly, or in whom latent TB was not detected were excluded.

Best corrected visual acuity was measured using Snellen’s chart and converted to logMAR (logarithm of the minimum angle of resolution) for statistical analysis. Anterior segment findings from slit-lamp examination and SC and MSC lesion status on fundus examination were evaluated. In addition, fundus photography, optical coherence tomography, fluorescein angiography, if available, indocyanine green angiography images were evaluated.

The patients were divided into two groups. Group 1 included 17 (12 MSC, 5 SC) patients treated with ATT and corticosteroid (CS) therapy. ATT consisted of a 4-drug regiment (300 mg/day isoniazid, 600 mg/day rifampicin, 2 mg/day pyrazinamide, and 1500 mg/day ethambutol) for 2 months, followed by a 2-drug regimen (300 mg/day isoniazid, 600 mg/day rifampicin) for 10 months. Treatments administered to group 1 patients during relapse are shown in Table 1.

Group 2 included 11 (9 MSC, 2 SC) patients treated with conventional IMT, interferon or CS monotherapy. Treatments administered to group 2 patients during relapse are shown in Table 2.

Visual acuities before treatment, at 1 year after treatment, and at final examination were compared between groups 1 and 2. The cumulative systemic CS dose received over the course of 1 year was calculated for both groups and compared.

Disease activity status, number of relapses, and remission time were calculated at the end of 1 year of treatment. The presence of active choroiditis was accepted as a criterion for activation. A patient was considered in remission when at least 1 year had elapsed since their last attack with no new choroiditis activation.

### Statistical Analysis

Fisher’s exact test was used to compare remission rates between groups; the Mann-Whitney U test was used to compare visual acuity and cumulative steroid dose between groups.

## RESULTS

A total of 21 MSC patients and 7 SC patients were included in the study. Mean age was 35.8±11.6 years for MSC patients and 44.6±12.8 for SC patients.

Group 1 consisted of a total of 29 eyes of 17 patients (11 male, 6 female) treated for 1 year with ATT + CS therapy. The patients’ mean age at presentation was 40.9±12.6 (28-64) years. Findings were consistent with MSC in 12 patients and SC in 5. Involvement was bilateral in 12 patients and unilateral in 5. Two of the SC patients had undergone 25 and 101 months of IMT prior to ATT, which was initiated due to reactivation, whereas ATT was the first choice for the other patients.

Group 2 included a total of 18 eyes of 11 patients (7 male, 4 female) treated for 1 year with IMT ± CS. The patients’ mean age at presentation was 33.4±10.8 (20-50) years. Findings were consistent with MSC in 9 patients and SC in 5. Involvement was bilateral in 7 patients and unilateral in 4. All patients in group 2 were treated with systemic CS; treatment was further supplemented with interferon alpha-2a in 2 patients, azathioprine in 2 patients, and combination azathioprine and cyclosporin therapy in 4 patients.

In group 1, clinical remission was observed in 12/12 MSC patients and 3/5 SC patients (total 15/17) after 1 year of ATT + CS therapy. Nine MSC and 1 SC patients remained in remission for 1-6 years after cessation of ATT (p=0.100). Reactivation occurred in 1 SC and 1 MSC patient at 2 and 7 months, respectively. Clinical remission was achieved in 2 SC patients resistant to ATT and two patients (1 SC, 1 MSC) with recurrence (Table 1).

In group 2, clinical remission was achieved in 7/9 MSC and 2/2 SC (total 9/11) patients after 1 year of IMT ± CS therapy, and remission continued for 1-3 years in 4 MSC patients and for 6-13 years in the 2 SC patients after cessation of treatment (p=0.454). Reactivation was observed in 2 MSC patients at 2 and 112 months, respectively. In 2 MSC patients with active disease at 1 year and 2 MSC patients with recurrence, remission was achieved with local/systemic CS and IMT modification (Table 2).

Comparison of treatment outcomes in MSC showed that remission was achieved in 9/12 MSC patients treated with ATT and 4/9 MSC patients treated with IMT (p=0.203). Among SC patients, only 1/5 treated with ATT and 2/2 treated with IMT achieved remission (p=0.142).

Median logMAR visual acuities at baseline, after 1 year of treatment, and at final examination were 0.4, 0.5, and 0.3 for group 1 and 0.8, 0.4, and 0.3 for group 2, respectively. There were no statistically significant differences between groups 1 and 2 in visual acuity at baseline and after 1 year of treatment (p=0.287).

After 1 year of treatment, the cumulative prednisolone equivalent mean CS dose was 1150±859 mg for group 1 and 1907±1979 mg for group 2 (p=0.057).

## DISCUSSION

The incidence of SC and MSC both in Turkey and worldwide has not been definitively determined. The largest case series study of MSC was conducted in 2003 by Gupta et al.^3^ in India, an endemic area for TB, and included 126 patients. Despite the paucity of epidemiological data regarding SC and MSC in Europe and America, studies conducted in these countries describe it as rare.^1,2,6,7,9^ Although the number of TB cases in Turkey dropped significantly toward the end of the 20^th^ century, there is still a higher incidence of latent TB compared to developed countries.^14^ Between 1995 and 2013, 63 patients were diagnosed with SC or MSC in our clinic. The higher prevalence of SC and MSC in India and Turkey may be related to the higher incidences of latent TB compared to developed countries. This supports the role of latent TB in the etiology of SC and MSC.

SC and MSC are generally considered to not show sex differences. However, Blumenkranz et al.^15^ first reported observing SC more often in males. Indeed, Gupta et al.^3^ reported that MSC was twice as common in males. In our study, males were predominant among both SC and MSC patients (male:female ratio=18:10).

In the literature, SC is reported to be most common in the white race, in the fourth and fifth decades, and is rare in young patients.^16^ However, studies conducted in India suggest that MSC is more common in younger individuals. The average age of MSC patients has been reported as 30 by Gupta et al.^3^ and 31 by Madhavan et al.^17^ Consistent with the literature, in the present study, the mean age of MSC patients was 35.8 years, while that in SC patients was 44.6 years. Furthermore, as in previous studies, most of the MSC and SC patients in our study exhibited bilateral involvement.

SC and MSC are clinical diagnoses based on typical fundus findings. The clinical morphology of MSC is unlike that of classic autoimmune SC, and it is believed to result from hypersensitivity to TB bacilli in patients with latent TB. The tuberculin skin test is the most commonly used test worldwide to detect latent TB. However, false-negative and false-positive results compromise the reliability of this test. Recent introduction of the more sensitive IGRA tests have substantially facilitated the diagnosis of latent TB. In the present study, IGRA was performed and positive results were confirmed for all patients in group 1 before initiating ATT. PPD was performed in 14/17 patients in that group, 3 of whom had indurations less than 15 mm. All patients with clinical suspicion of MSC who underwent IGRA had positive results. Studies conducted by the manufacturer report the reliability of IGRA results as being in the 90-95% range.^18^ Although we found a 100% positivity rate among our patients, Mackensen et al.^9^ reported a very low IGRA accuracy rate for MSC (52%). In the same study, 4 patients remained IGRA positive after ATT. This indicates that the IGRA cannot be used in patient follow-up.

Like the IGRA, polymerase chain reaction (PCR) analysis is another new diagnostic method for MSC. Gupta et al.^3^ conducted PCR analysis on aqueous and vitreous samples from 7 MSC patients and reported that results were positive for TB in 5 (71.4%) of them. We did not conduct PCR analysis for any of our patients in the present study.

Although the diagnosis of SC and MSC are based on clinical data, treatment is a controversial topic among the global scientific community. In countries not endemic for TB, SC is considered an autoimmune-derived disease, and treatment with immunosuppressive agents is recommended. Particularly in the presence of clinically active choroiditis, the current therapeutic approach includes high-dose CS therapy, followed by IMT to prevent recurrence in the long term.^19^ There is no consensus in the literature regarding which immunomodulatory agents are more effective in the treatment of SC. T-cell inhibitors, antimetabolites, alkylating agents, and biologic agents are the different treatment alternatives currently in use.

In the current study, the SC patients in groups 1 and 2 showed contradictory treatment responses. Five patients in group 1 who had findings consistent with classic SC and latent TB according to IGRA were treated with ATT ± CS; of these, 2 patients continued to have clinically active disease after treatment, and 1 patient showed reactivation 2 months after treatment cessation. The poor treatment results in 3 of the 5 patients treated with ATT may be a coincidence related to the high rate of latent TB in the Turkish population. On the other hand, in the 2 patients treated successfully with ATT, the disease could not be controlled previously with IMT + CS therapy. This suggests that the choroiditis may be associated with latent TB. In group 2, which was not treated with ATT, 2 patients diagnosed with classic SC were treated with IMT + CS therapy. Both of those patients were in clinical remission after 1 year of treatment, and no relapse was observed during follow-up. This contradiction raises the question of whether ATT should be initiated when latent TB is detected in patients with classic SC.

Studies conducted in India have reported that ATT effectively reduces attacks and induces long-term remission in patients with tuberculosis-associated MSC.^20^ In their study conducted in Germany, Mackensen et al.^9^ also reported observing remission without relapses in MSC patients treated with ATT. Similarly, in the present study we achieved remission in a majority of MSC patients after 1 year of ATT ± CS therapy. Only 1 patient experienced relapse after 7 months. Of the MSC patients treated with IMT ± CS, 2 patients had persistent clinical activity after 1 year of treatment, and 2 others relapsed during follow-up. Based on these results, we can conclude that ATT is more effective in MSC patients. Furthermore, the lower cumulative CS dose in group 1 also demonstrates the efficacy of ATT. Patients in group 2 required CS at higher doses and for longer periods to control active inflammation.

## CONCLUSION

The etiopathogenesis and treatment of SC and MSC remain controversial. Although SC is described in the literature as an autoimmune disease, MSC is reportedly associated with TB. Latent TB is common among Turkish patients, and the possibility of also detecting latent TB in SC patients poses a problem in terms of treatment approach. Although statistically significant results could not be obtained due to small patient numbers, our observation that ATT may be more effective in the treatment of MSC is consistent with the literature. Prospective studies with larger case series are necessary.

## Figures and Tables

**Table 1:**
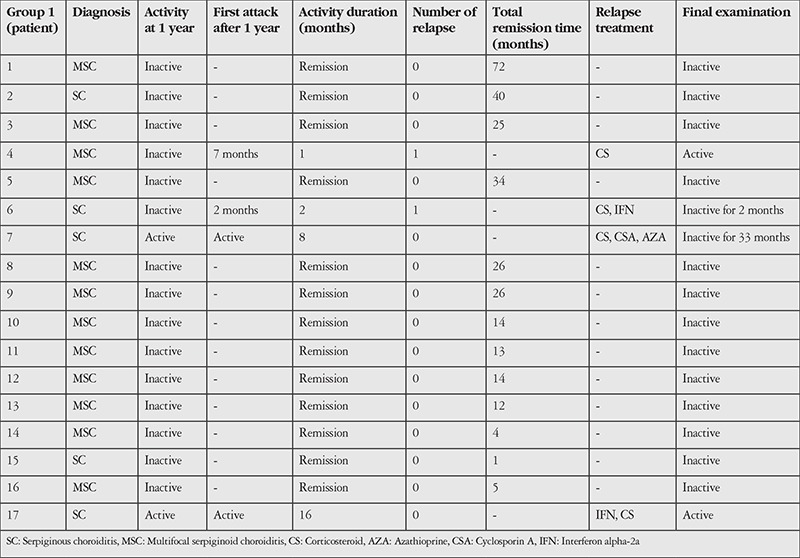
Status of group 1 patients after 1 year of treatment and during follow-up, and therapy added for relapse

**Table 2:**
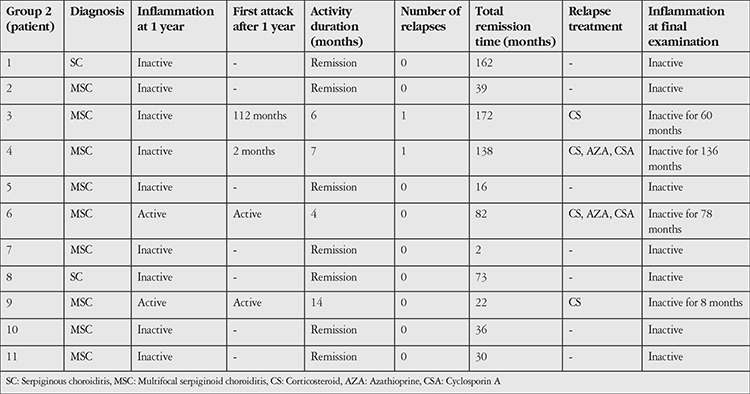
Status of group 2 patients after 1 year of treatment and during follow-up, and therapy added for relapse
